# Room-Temperature Luminescence of Eosin Y and Phloxine B in Red- to Near-Infrared Optical Region

**DOI:** 10.1007/s10895-026-04822-4

**Published:** 2026-06-19

**Authors:** R. Max Petty, Bong Lee, Agnieszka Jablonska, Rajveer Sagoo, Danh Pham, Trang Thien Pham, Sergei V. Dzyuba, Zygmunt Gryczynski, Ignacy Gryczynski

**Affiliations:** 1 Department of Physics and Astronomy, Texas Christian University, Fort Worth, TX 76129 USA; 2 Department of Chemistry and Biochemistry, Texas Christian University, Fort Worth, TX 76129 USA

**Keywords:** Eosin Y, Phloxine B, Delayed fluorescence, Phosphorescence, Room-temperature luminescence, Polyvinyl alcohol

## Abstract

Room temperature luminescence of two far-red emitting dyes, Eosin Y and Phloxine B, embedded in polyvinyl alcohol films have been studied. Three types of luminescence emission, including prompt fluorescence, delayed fluorescence, and phosphorescence have been characterized. Fluorescence and delayed fluorescence of Eosin Y and Phloxine B appear in the orange-red optical region, whereas phosphorescence emissions cover far-red and near infra-red (NIR) spectral regions reaching over 800 nm. The millisecond phosphorescence lifetimes, high relative brightness of Eosin Y and Phloxine B, and unusually high phosphorescence anisotropy make them attractive optical room-temperature phosphorescence (RTP) materials in red to NIR spectral regions.

**Supplementary Information:**

The online version contains supplementary material available at 10.1007/s10895-026-04822-4.

## Introduction

Room temperature phosphorescence (RTP) of purely organic materials is one of the most interesting and potentially useful phenomena in spectroscopy and material science areas, due to promising applications in optical sensing [1–7], encryption-decryption [8–12], anti-forgery [13–16], light emitting diodes [17–20], dye-polymer matrix interactions [21–24], and resonance energy transfer processes [25–28]. The RTP systems include dyes in a crystalline state [29–32], isolated on filter paper [33, 34], encapsulated in nano/micro particles [35], or embedded in polymer matrices [4, 36, 37]. Polyvinyl alcohol (PVA) is commonly used as a matrix for RTP because its dense hydrogen-bonded network limits oxygen diffusion and is relatively easy to prepare. Notably, the majority of PVAs are water soluble, which offers the possibility to adjust pH of the polymer-containing medium during the film formation process [36]. The reports on RTP systems that cover blue-green-yellow regions of the optical spectrum are abundant, yet RTP in red/near-infrared (NIR) is less represented. Although xanthene red dyes are known for their delayed fluorescence (DF) and phosphorescence (PH), they were not systematically studied from a RTP perspective. In this report, we present a comprehensive study of room temperature luminescence properties of Eosin Y (Eos) and Phloxine B (Phlox) embedded in PVA films that cover red and far-red spectral regions. These dyes exhibit E-type delayed fluorescence, also known as thermally activated delayed fluorescence (TADF). Thermal repopulation of the singlet excited state (S_1_) from the triplet state (T_1_) is possible because these states are in thermal equilibrium, and the local temperature in the vicinity of an excited molecule (on the order of milliseconds) is sufficiently high enough to overcome the singlet-triplet energy gap (about 3700 cm^− 1^ and 3400 cm^− 1^ for Eosin and Phloxine, respectively). The relative intensities of E-type delayed fluorescence and phosphorescence in rigid PVA films are temperature dependent, as has been shown in previous literature [37, 38].

## Materials and methods

Polyvinyl alcohol (MW 130,000; 98% hydrolyzed), Eosin Y (dye content 99%) and Phloxine B (dye content ≥ 80%) were obtained from Sigma-Aldrich, and they were used as received.

### PVA Film Preparation

The PVA films with and without Eos and Phlox were made from PVA-containing solutions following literature procedures [37]. Dye concentrations in PVA films were estimated using extinction coefficients and measured film thicknesses, and independently assumed a reduction in volume by 12.5-fold upon drying [37]. To minimize possible dimer formation and aggregation in PVA films, three progressively higher concentrations were prepared and their absorbances evaluated. The dried PVA films were stored in a desiccator before measurements were taken. For the measurements, PVA film strips were placed in a 1 mm thick micro cuvette and filled with benzene, which is inert to PVA and prevents humidity from affecting the sample film.

### Absorption Measurements

Absorption spectra were collected using the Varian Cary 60 UV-Vis spectrophotometer (Agilent Technologies, Inc., Santa Clara, CA, USA). The PVA film has been used as the baseline blank. It should be noted that at the red spectral region, the absorption of the PVA film is negligible.

### Steady-State Fluorescence Measurements

Fluorescence spectra were collected with Varian Cary Eclipse spectrofluorometer (Agilent Technologies, Inc., Santa Clara, CA, USA). The format of front-face geometry was used to conduct steady-state fluorescence measurements with custom-made attachments [37].

### Phosphorescence Spectra Measurements

Phosphorescence excitation and emission spectra were measured using the time-gated phosphorescence detection mode of the Varian Cary Eclipse. The following parameters were used unless otherwise specified: Total Decay Time—0.05s, Number of flashes—10, Delay Time—0.1 ms, and Gate Time—5 ms. These parameters were able to remove short-lived emission components such as prompt fluorescence and scattering.

### Luminescence Anisotropy

For luminescence anisotropy measurements, the Varian Cary Eclipse spectrofluorometer was fitted with grid polarizers on both excitation and on observation paths. This was necessary because commonly used sheet polarizers are not adequate for far-red spectral region measurements. Anisotropies were calculated using the following equation:

1 $$r=\frac{I_{VV}-GI_{VH}}{I_{VV}+2GI_{VH}}$$

where the $\:{\mathrm{I}}_{\mathrm{V}\mathrm{V}}$ and $\:{\mathrm{I}}_{\mathrm{V}\mathrm{H}}$ components are the fluorescence intensities when excited with vertical polarization (V) and observed with horizontal (H) and vertical (V) polarization, respectively. The G-Factor, represented as $\:\mathrm{G}$, was used to compensate for the uneven transmission of different polarizations through the detection path.

### Luminescence Lifetime Measurements

#### Fluorescence Lifetime Measurements

Fluorescence lifetimes were measured with FT200 fluorometer (PicoQuant PicoQuant, GmBH) using 485 nm picosecond pulsed laser diode on the excitation. The collected decay profiles were analyzed using FluoFit4 (PicoQuant GmBH, Berlin, Germany), which provides decay analysis along with standard deviations and confidence intervals of recovered parameters.

The lifetime decays were fit to the following model:

2 $$I(t)=\int_{-\infty}^tIRF(t')\sum_ia_ie^{\textstyle\frac{-(t-t')}{\tau_i}}$$

Equation (2) represents a deconvolution model where IRF(t’) is the Instrument Response Function at t = t’. The $\:{\alpha\:}_{i}$ term represents the amplitude for the i^th^ intensity decay component at a time to and $\:{\tau\:}_{i}$ represents the lifetime of that component.

#### Delayed Fluorescence and Phosphorescence Lifetime

Delayed fluorescence and phosphorescence lifetime measurements were performed using the Varian Cary Eclipse spectrofluorometer, which is equipped with a phosphorescence lifetime mode capable of measuring lifetimes in a millisecond time scale. The same parameters were used as in the steady-state mode (see Sect. 2.4.) except for gating, which was set to 0.5 ms. The Varian Eclipse lifetime program provides one- and two-exponential decay analysis and estimates standard deviations of the recovered parameters.

### Fluorescence Quantum Yield Measurement

The fluorescence quantum yield (QY) was estimated by comparison to reference fluorescence of Rhodamin 6G (R6G) in ethanol using 1 mm pathlength cuvettes. The cuvette with a dye-doped PVA strip was filled with benzene. This solvent is inert to PVA and perfectly matches its refractive index causing the PVA strip to become invisible (see Figure SM1 in *Supplementary Materials*). The QY was calculated follows:

3 $$Q_S=Q_R\frac{I_S\left(1-10^{-A_R}\right)n_S^2}{I_R\left(1-10^{-A_S}\right)n_R^2}$$

Where, $\:{Q}_{S}$ is the QY the sample measured,$\:{Q}_{R}$ is QY of the Rhodamin 6G (R6G) in ethanol ($\:{Q}_{R}=$0.95), $\:{I}_{R,S}$ is the total integrated intensity, $\:\:\:{A}_{R,S}$ is the absorbance at the excitation wavelength,, $\:\:\:{n}_{S}$ is the refractive index of benzene/PVA (1.48), and $\:{n}_{R}$ is the refractive index of ethanol (1.36). The subscripts *R*,* S* stand for reference and sample respectively.

## Results and Discussion

This section provides a comprehensive study of the spectral properties of the investigated dyes. First, absorptions and prompt fluorescence spectra are described, followed by anisotropy and lifetime measurements. Next, excitation and emission spectra of RTP are presented, followed by RTP anisotropy and lifetime measurements.

### Absorption and Fluorescence Spectra

**Fig. 1:**
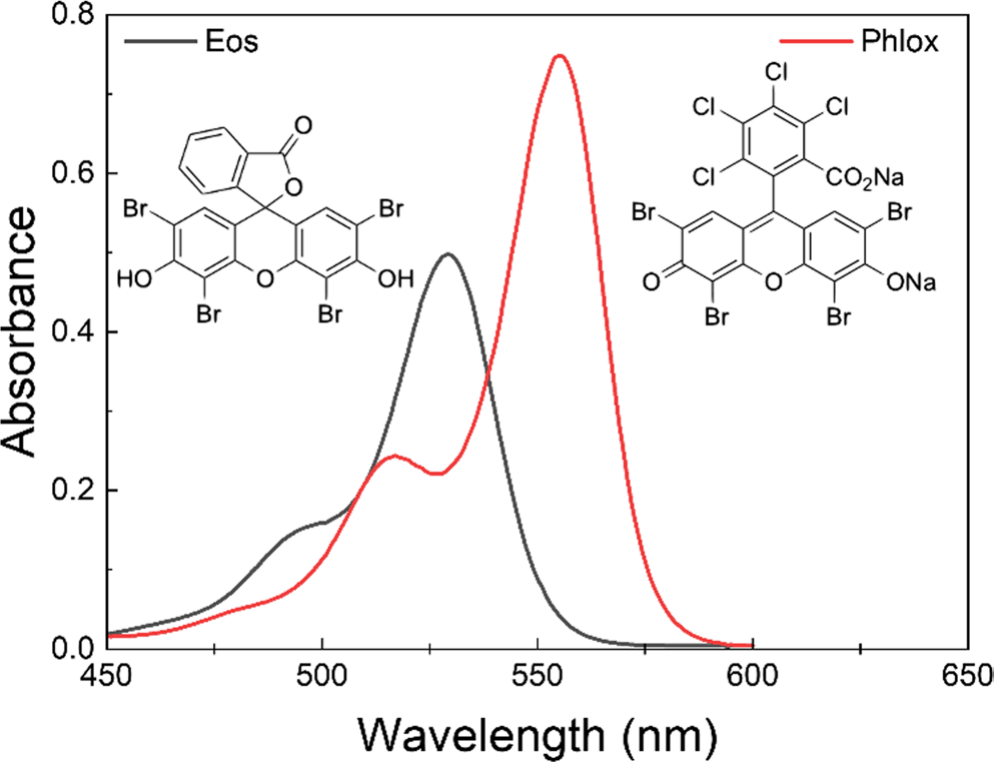
Absorption spectra of Eos (black) and Phlox (red) in PVA films. The molecular structure of each dye are shown next to the spectra (for other possible forms of these dyes, see Chart 1 in *Supplementary Materials*)

Both dye-doped PVA samples exhibit bright and spectrally distinct fluorescence (orange for Eos and red for Phlox) under UV/visible light excitation, confirming that the PVA matrix effectively stabilizes the dyes in an emissive state at room temperature (Figure SM2, see *Supplementary Materials*). Absorption spectra of Eos and Phlox in PVA films are shown in Fig. 1. The maxima are centered at 529 nm (0.498) and 555 nm (0.749) for Eos and Phlox, respectively. Taking into account extinction coefficients of the compounds (120,000 for Eos and 83,000 for Phlox) (PhotochemCAD) and a thickness of the PVA polymer film of 490 μm (as measured with a caliper), we estimated concentrations of the dyes as 83µM for Eos and 180µM for Phlox, respectively. From a series of films prepared at different concentrations (see Figure SM3 in the *Supplementary Materials*), those with intermediate absorbance values were selected for further analysis. The absorption spectral shapes of both Eos and Phlox remained unchanged with concentration, indicating the absence of dimer or aggregate formation within the investigated concentration range.

**Fig. 2:**
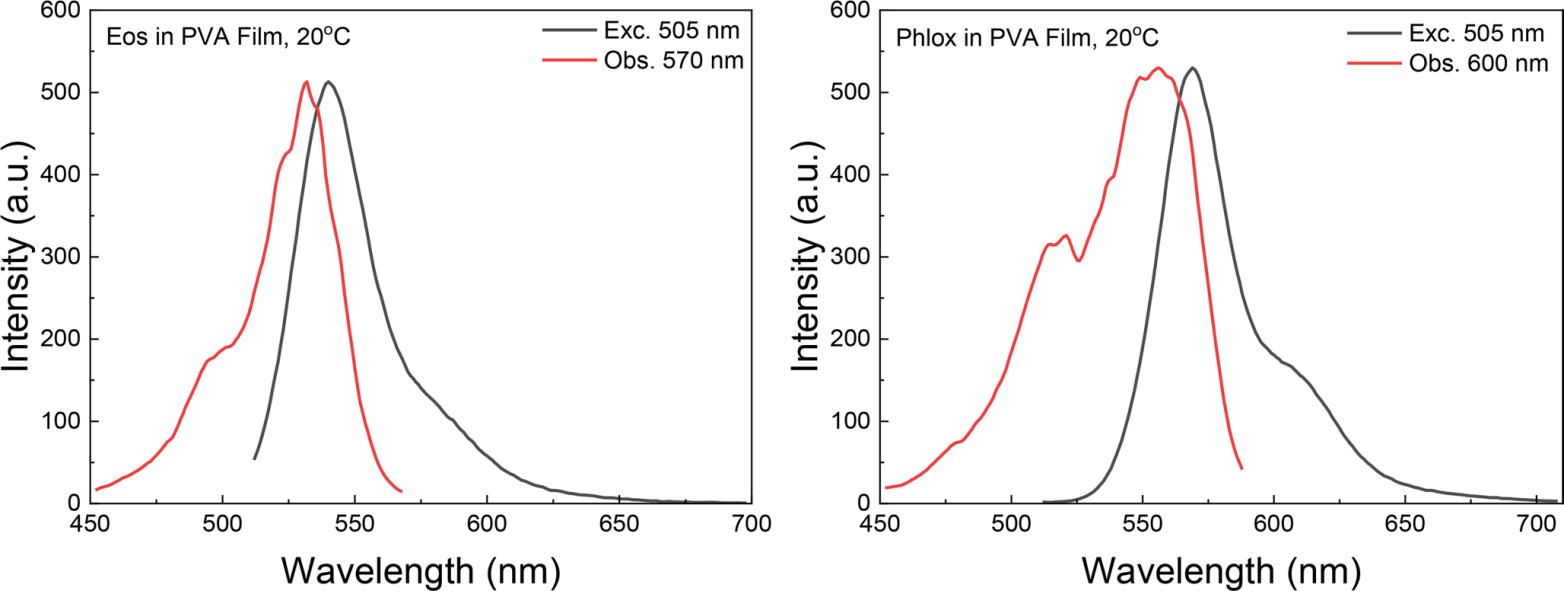
Fluorescence emission and excitation spectra of Eos (left) and Phlox (right) embedded in PVA films. The exitation spectra were recorded at 570 nm (Eos) and 600 nm (Phlox). Fluorescence emission spectra were collected upon excitation at 505 nm

Fluorescence spectra are shown in Fig. 2. The excitation spectra resembles the dye absorption spectra presented in Fig. 1. The fluorescence emissions spread from 500 nm to 650 nm for Eos-doped PVA film and from 525 nm to 675 nm for Phlox-doped PVA film. The fluorescence maxima are centered at 540 nm for Eos and 570 nm for Phlox.

The fluorescence excitation and emission spectra exhibit small Stokes shifts and partial mirror symmetry.

### Fluorescence Anisotropy

**Fig. 3:**
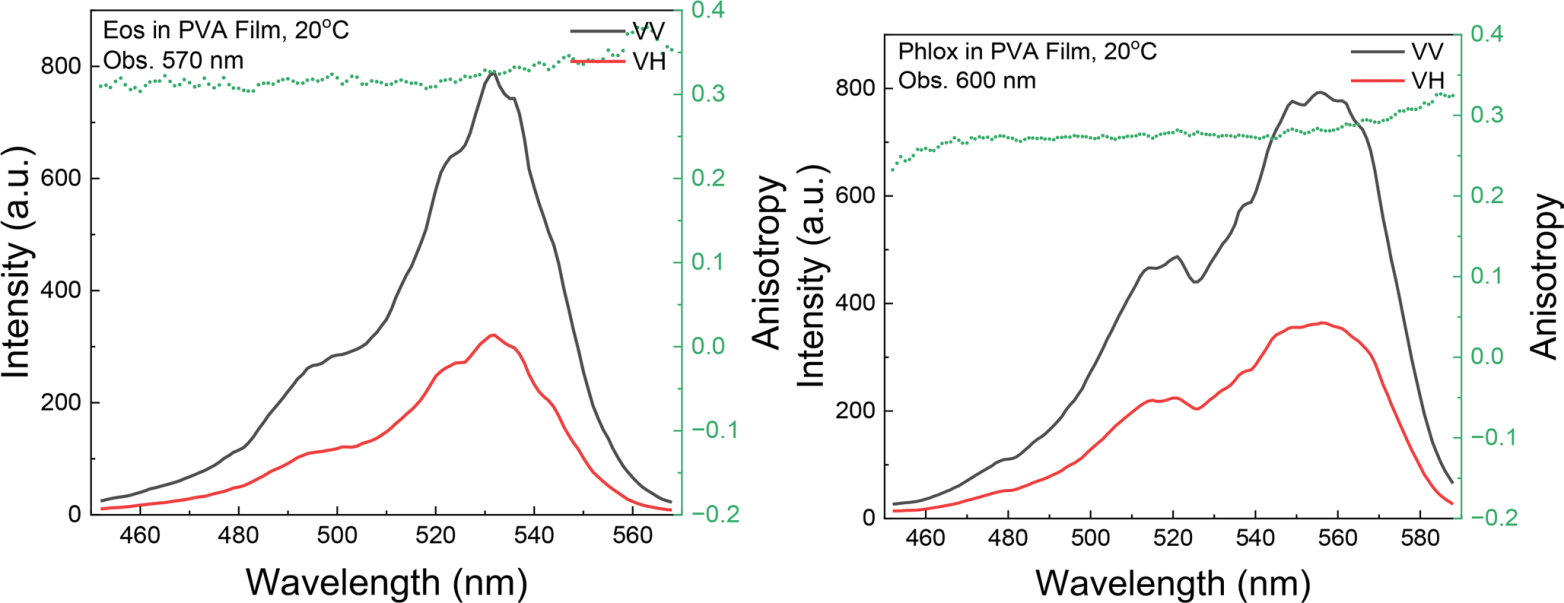
Fluorescence excitation anisotropies of Eos (left) recorded with observation at 570 nm and Phlox (right) with observation at 600 nm, both in PVA films. The anisotropies were calculated from polarized components VV and VH

During PVA film formation, dye molecules are immobilized within the polymer matrix, restricting their translational and rotational degrees of freedom. Consequently, fluorescence emission is expected to be polarized due to photoselection effect. The measured excitation anisotropy values are approximately 0.3 and remain uniform across the entire excitation spectra for both dyes (Fig. 3). This high value of fluorescence anisotropy show that fluorophores are effectively immobilized within the polymer matrix. In addition, this also suggests that the excitation occurs within a single electronic transition since the transition moments for excitation to higher electronic states usually have different directions as compared to S_0_-S_1_ transition resulting in lower anisotropy.

**Fig. 4:**
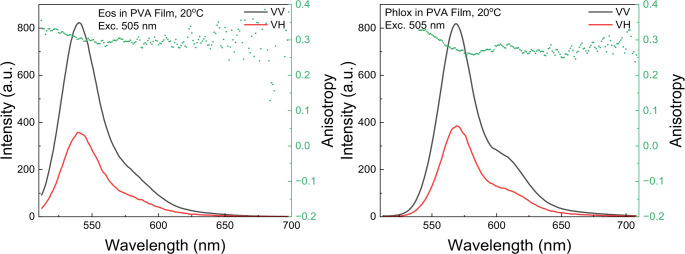
Fluorescence emission anisotropies of Eos (left) and Phlox (right) in PVA films, recorded upon excitation at 505 nm

The fluorescence emission anisotropies are slightly decreased at longer wavelengths (Fig. 4). Such decreases are often observed and are attributed to the spectral relaxation. Although dye rotations are suppressed in a rigid polymer, the restricted tortional movements of functional groups could be possible, which, in turn, may slightly decrease the observed anisotropies.

The fluorescence anisotropy is sensitive to the concentration of dyes. At higher concentrations, the excitation energy can be transferred by Förster mechanisms, either Förster resonance energy transfer (FRET) between different dyes or homo-FRET between identical dye molecules, to nearby unexcited molecule, allowing excitation energy to migrate between molecules. As a result, fluorescence emitted from molecules other than the originally excited molecule can become depolarized. To avoid concentration-dependent depolarization, low dye concentrations should be used. We measured fluorescence anisotropy in Eos- and Phlox -doped PVA films at approximately half of the original concentrations (about 40µM for Eos and 90µM for Phlox) and obtained the same results as when compared to the original films. Therefore, we conclude that energy migration is not significant at the concentrations used.

### Fluorescence Lifetime

**Fig. 5:**
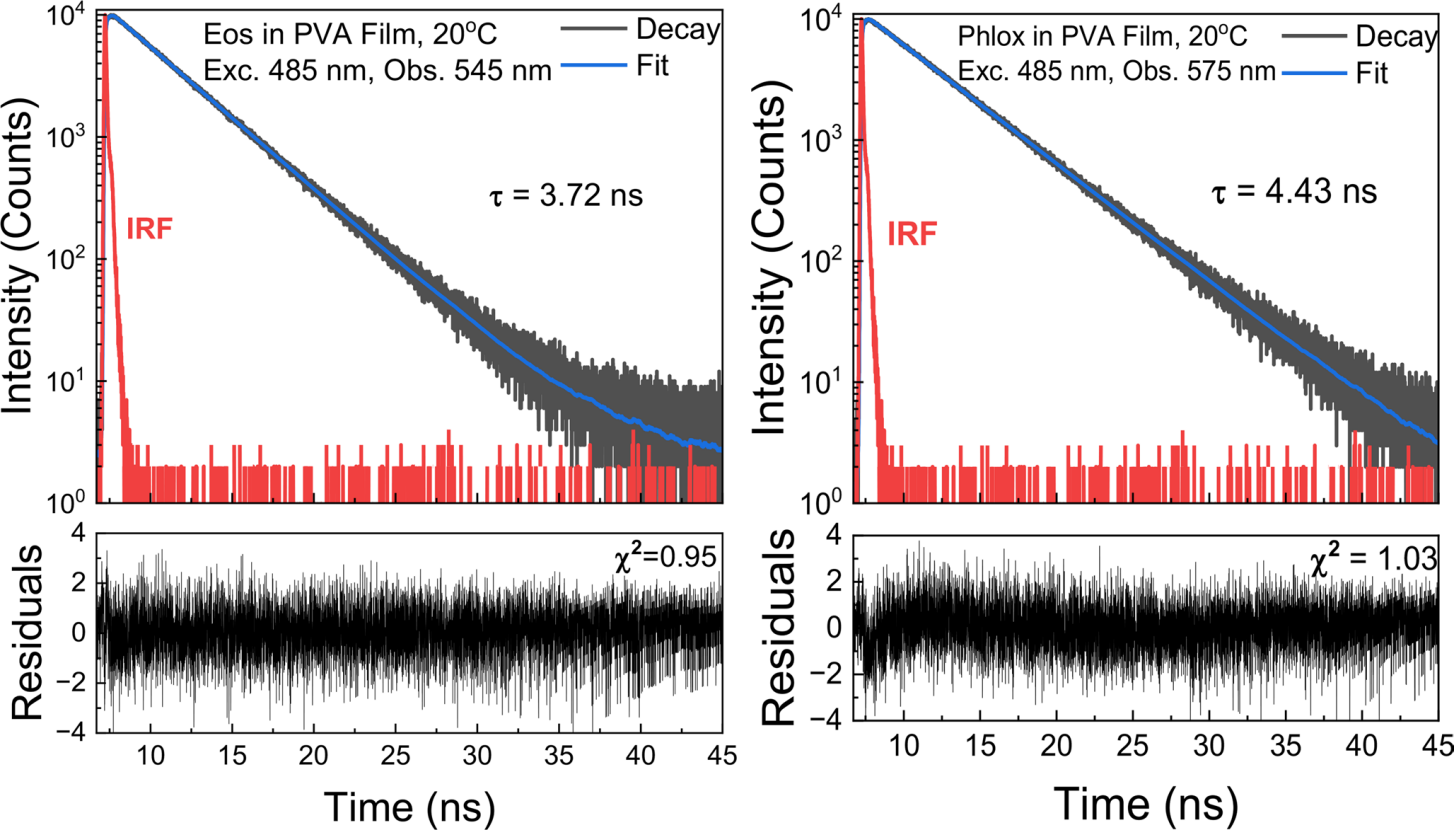
Fluorescence lifetimes of Eos (left) and Phlox (right) in PVA films. The fluorescence intensity decays are well described by a single-exponential model. The recovered lifetimes exhibit high accuracy with low standard deviations (0.02 ns for Eos and 0.03 ns for Phlox)

Fluorescence intensity decays of both samples are shown in Fig. 5. Both Eos and Phlox samples show decays which can be well fitted with a single exponent. The significantly extended fluorescence lifetimes in PVA films, compared to solution, highlight the role of the matrix rigidity in suppressing non-radiative decay and enhancing excited-state stability.

### Fluorescence Quantum Yield

**Fig. 6:**
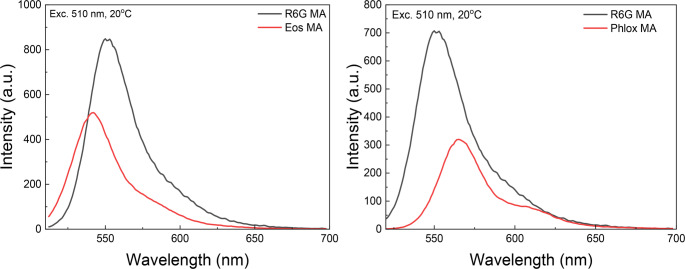
Comparison of fluorescence emission spectra of Eos (left) and Phlox (right) in PVA films, recorded upon excitation at 510 nm, with Rhodamine 6G in ethanol. The corresponding absorption spectra of the samples are in Figure SM4 in *Supplementary Materials*

Fluorescence QY of Rhodamine 6G (R6G) in ethanol is a well-established reference standard, established using different methods (optical, photoacoustic or thermal lens spectroscopy) with a value of around 0.95 [39–42]. This value has been used to determine the relative QY of Eos and Phlox in PVA films. The comparison of Eos and Phlox fluorescence spectra with the R6G in ethanol (measured as described in Materials and Methods section) is shown in Fig. 6. At the excitation wavelength of 510 nm, all three samples have the same absorption, see Figure SM3 in *Supplementary Materials.* The estimated QYs values are 0.64 (± 0.02) and 0.43 (± 0.02) for Eos and Phlox-doped PVA films, respectively. The QYs in PVA are higher than in the water solutions because diffusion-dependent quenching processes are knock down.

### Room-temperature Delayed Fluorescence and Phosphorescence

####  Excitation and Emission Spectra

**Fig. 7:**
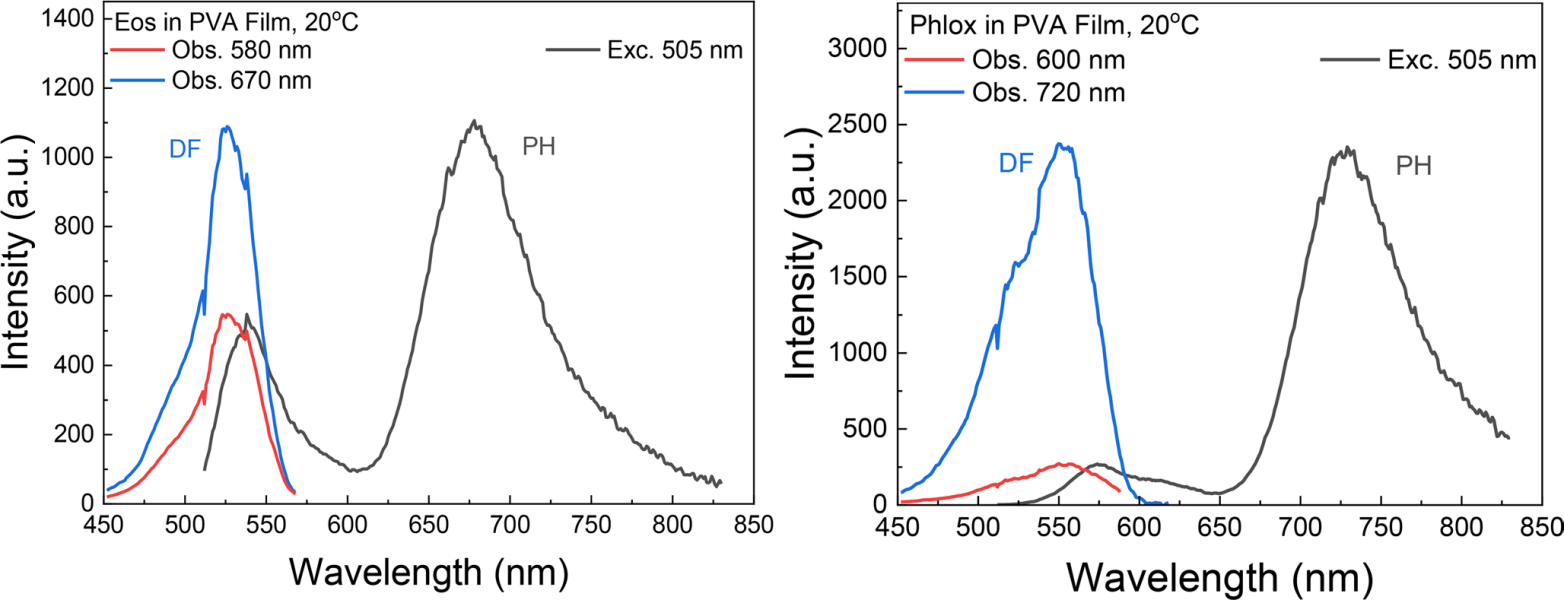
Delayed Fluorescence and Phosphorescence excitation and emission spectra of Eos (left) and Phlox (right) in PVA films

Luminescence measurements with gated detection (see Materials and Methods section) eliminate prompt processes, such as fluorescence and scattering, by collecting photons after a delay of 100 µs. Therefore, with the gated detection (Phosphorescence Mode) only DF and PH are detected. These excitation and emission spectra are shown in Fig. 7.

DF and PH spectra of both dyes are separated by more than 100 nm, enabling unperturbed spectral measurements. The excitation spectra (red lines for DF and blue lines for PH) mimic the absorptions (Fig. 1) and excitation spectra of prompt fluorescence (see Fig. 2). This indicates that PH is populated via the singlet S_1_ state. The thermal dependence of the DF/PH ratio has been previously investigated [37, 38] and demonstrated that DF is a thermally activated process (TADF).

#### Delayed Fluorescence and Phosphorescence Anisotropies

**Fig. 8:**
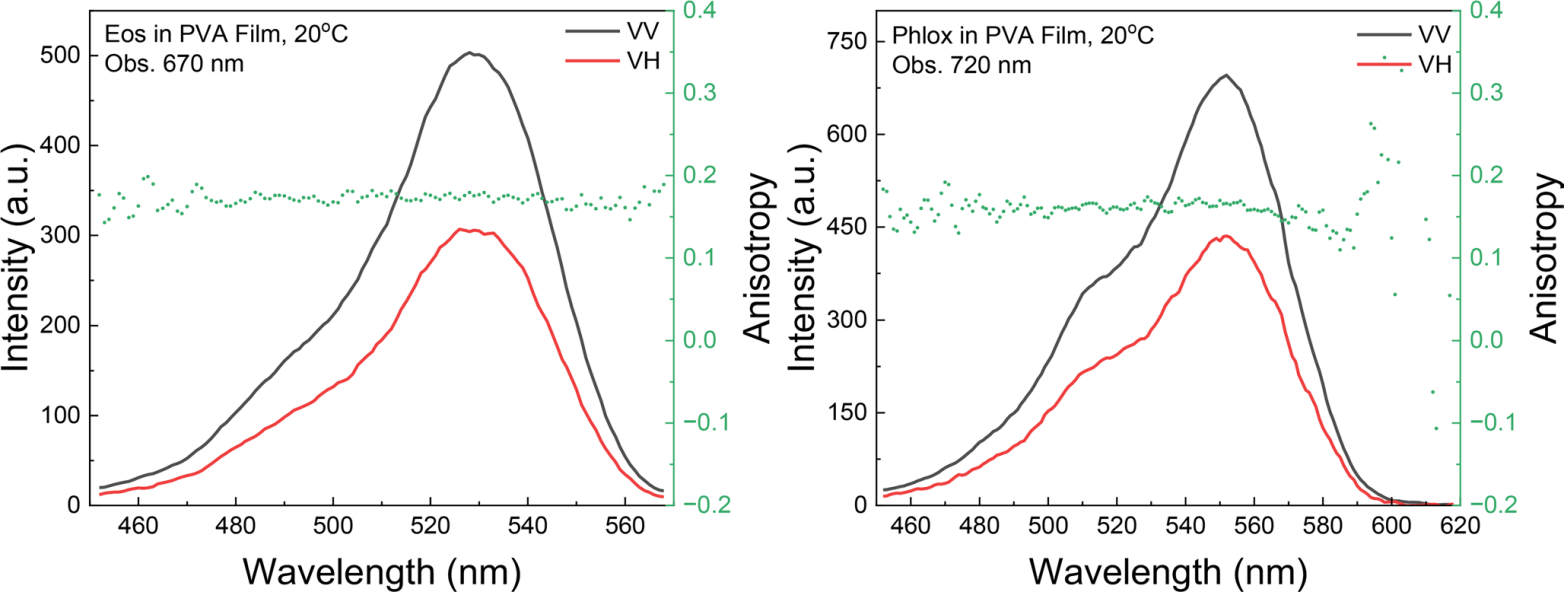
Phosphorescence excitation anisotropies of Eos (left) recorded with observation at 670 nm, and Phlox (right) with observation at 720 nm, both in PVA films

The PH excitation anisotropies remain constant across the excitation wavelength range (Fig. 8) indicating that, for both Eos and Phlox, excitation occurs predominantly into a single electronic state (S_1_). Similarly, the DF excitation anisotropies are also wavelength-independent (Figure SM5 in *Supplementary Materials*).

**Fig. 9:**
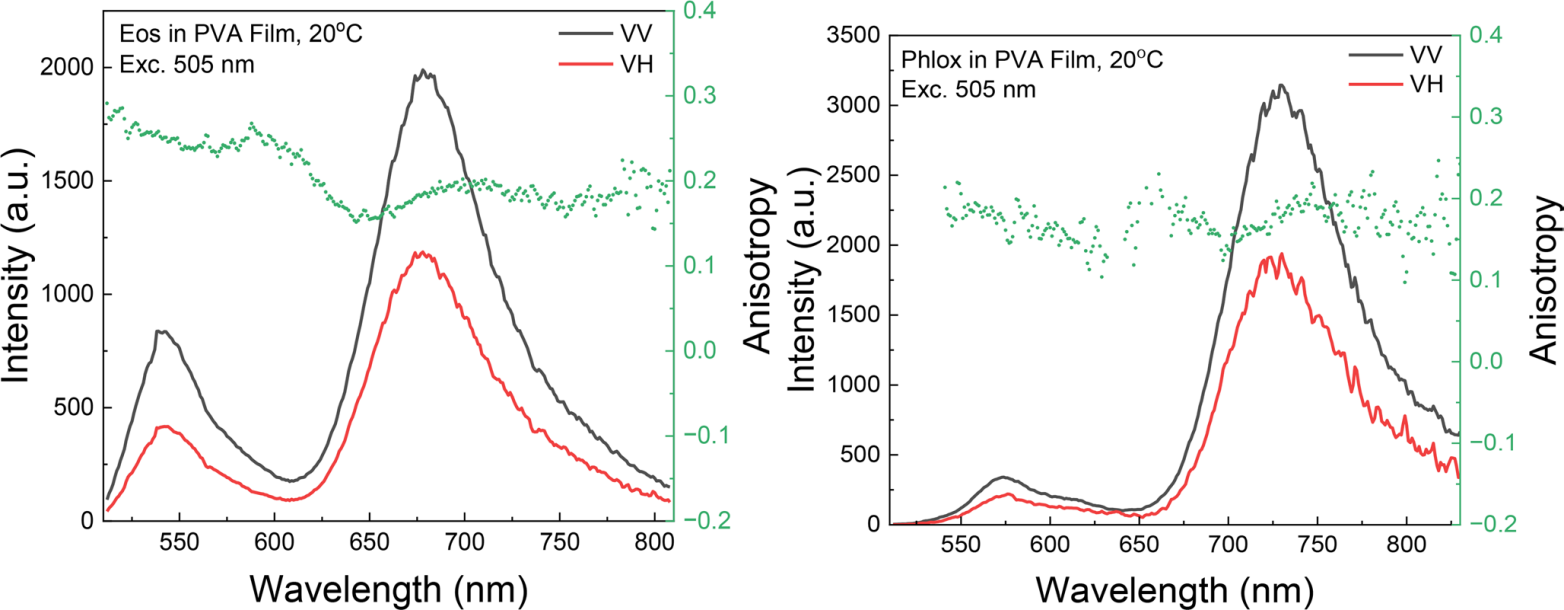
Phosphorescence emission anisotropies of Eos (left) and Phlox (right) in PVA films, recorded upon excitation at 505 nm

The PH emission anisotropy (Fig. 9, left) show relatively high values. In the Eos-doped PVA film, the DF anisotropy is only slightly lower than fluorescence anisotropy (Fig. 4), while the PH anisotropy is approximately 0.2. In contrast, for Phlox, the DF and PH anisotropies are identical (Fig. 9, right).

#### Delayed Fluorescence and Phosphorescence Lifetimes

**Fig. 10:**
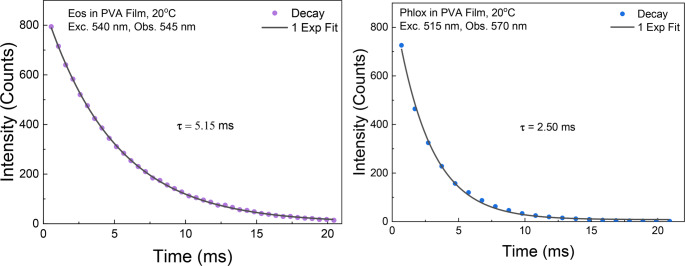
Delayed Fluorescence intensity decays of Eos (left), recorded with excitation at 540 nm and observation at 545 nm, and Phlox (right), recorded with excitation at 515 nm and observation at 570 nm, both in PVA films

**Fig. 11:**
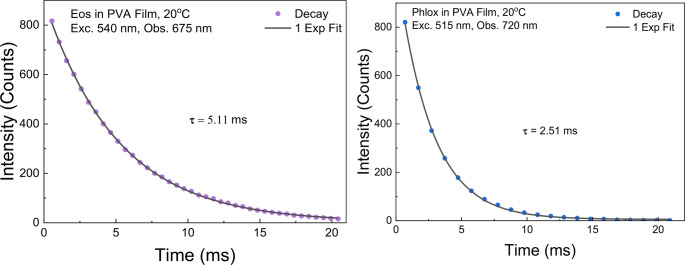
Phosphorescence intensity decays of Eos (left), recorded with excitation at 540 nm and observation at 675 nm, and Phlox (right), recorded with excitation at 515 nm and observation at 720 nm, both in PVA films. The standard deviations of the recovered lifetimes were 0.05 ms for Eos and 0.04 ms for Phlox

The DF and RTP lifetimes of Eos- and Phlox-doped PVA films are in the single-digit millisecond region. Both, DF and RTP display similar lifetimes for both compounds, 5.1 ms for Eos and 2.5 ms for Phlox (Figs. 10 and 11).

Time-dependent DF and/or RTP intensities can be well fitted with a single exponential function. (solid lines in Figs. 10 and 11).

#### Relative Intensity of Room-Temperature Phosphorescence

**Fig. 12:**
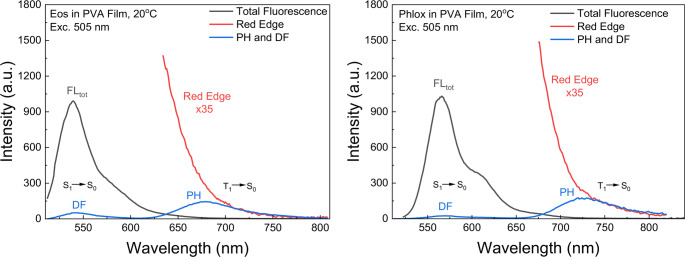
Full-range emission spectra and red-edge of Eos (left) and Phlox (right) in PVA Film. The DF and PH spectra were constructed under the assumption that the long-wavelength portion of red-edge spectra (above 700 nm for Eos and 730 nm for Phlox) does not contain a fluorescence contribution

In low-temperature luminescence measurements, where PH can be efficienty detected without time-gating acquisition, efficiences of fluorescence and PH can be determined precisely. In the case of RTP, however, the situation is more complex because the TADF is no longer negligible. Although RTP and TADF appear strong under gated detection, fluorescence overwhelmingly dominates the observed spectrum under steady-state conditions (i.e. without detection delay) (Fig. 12). Below we present an attempt to describe relative RTP in comparison to fluorescence. Full emission spectra were recorded using 35-fold attenuation (neutral density filters in the detection path), whereas the red-edge emission was recorded without attenuation. These red-edge spectra contain strongly overlapping contributions from both fluorescence and RTP. Based on the assumption that emission above 720 nm for Eos and 750 nm for Phlox is dominated by PH, we reconstructed PH spectra (blue lines in Fig. 12). Conservatively, we estimated the fractions of RTP in the entire emission as 0.67% and 0.74% for Eos-doped PVA film and Phlox-doped PVA film, respectively. In conclusion, the quantum yields of Eos-doped PVA film and Phlox-doped PVA film are equal or below 0.004 and 0.003, respectively. Only upper limits of the QYs can be estimated, as fluorescence photons in the PH spectral region cannot be completely excluded. Despite these relatively low QYs, RTP is easily detectable because prompt fluorescence and scattering are effectively eliminated by gated detection.

## Conclusions

The room-temperature luminescence properties of two red xanthene dyes, Eosin Y and Phloxine B, embedded in PVA films have been characterized. Both dyes exhibit bright fluorescence emissions with relatively high fluorescence quantum yields of 0.64 and 0.43 for Eos and Phlox, respectively. The high fluorescence anisotropies indicate that dyes are strongle immobilized within the PVA polymer matrix. Remarkably, both systems also display bright RTP with millisecond lifetimes. The PH emission of Eos-doped PVA films spans the 600–800 nm range, while that of Phlox-doped PVA films extends from 650 to 850 nm. Consequently, a mixture of the two dyes produces broadband RTP covering the 600–850 nm spectral range (Figure SM6 in *Supplementary Materials*). The PH anisotropies of both dyes are high, reaching 0.25 for Eos and nearly 0.2 for Phlox. This observation is unexpected, as PH anisotropies are typically very low or even negative unless the triplet state is populated via direct excitation [43–46]. With the intersystem crossing involved, the PH transition dipole moment is expected to be orthogonal to that of fluorescence, with limiting anisotropy of −0.2. The anisotropy experiments were repeated for several independently prepared films, yielding consistent results. At present, we are unable to provide a theoretical framework for the observed PH anisotropies.

Nevertheless, these findings may be of interest for studies of macromolecular dynamics. The high anisotropies observed for Eos and Phlox may be advantageous for anisotropy-based imaging applications [47]. Collectively, these findings establish Eosin Y and Phloxine B as promising organic materials for red and NIR room-temperature phosphorescence, with potential relevance to time-resolved phosphorescence spectroscopy. Recently, secret writing and anti-counterfeiting technologies have attracted significant interest [13, 8, 48], and we believe the proposed dyes could be useful in these applications. Finally, because materials for organic light-emitting devices (OLEDs) are predominantly limited to the green spectral region, new blue, red, and NIR optical materials are still needed, highlighting the broader relevance of this work [49, 50].

## Supplementary Information

Below is the link to the electronic supplementary material.

Supplementary Material 1 (DOCX 730 KB)

## Data Availability

The original contributions presented in this study are included in the article/supplementary material. Further inquiries can be directed to the corresponding author(s).

## Abbreviations

Eos: Eosin Y
Phlox: Phloxin B
PVA: Polyvinyl Alcohol
QY: Quantum Yield
RTP: Room Temperature Phosphorescence
DF: Delayed Fluorescence
PH: Phosphorescence
TADF: Thermally Activated Delayed Fluorescence
