# Supplementary material for: Room-Temperature Luminescence of Eosin Y and Phloxine B in Red- to Near-Infrared Optical Region
Source: J Fluoresc. 2026 Jun 19;36(6):4155–65. doi: 10.1007/s10895-026-04822-4 (PMC13331955; doi:10.1007/s10895-026-04822-4)
Supplement: Supplementary file 1 — Supplementary Material 1 (DOCX 730 KB) [file 10895_2026_4822_MOESM1_ESM.docx]

# **Supplementary Materials**

# In the index matching fluid (benzene), the PVA film is practically invisible.

# **
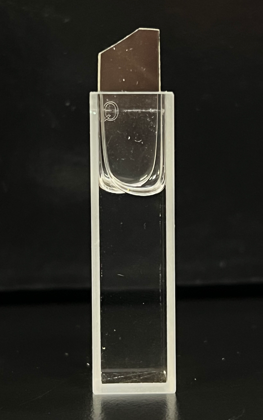
**

# **Figure SM1.** Photograph of microcuvette containing a PVA strip with an index-matching fluid (benzene).

# **
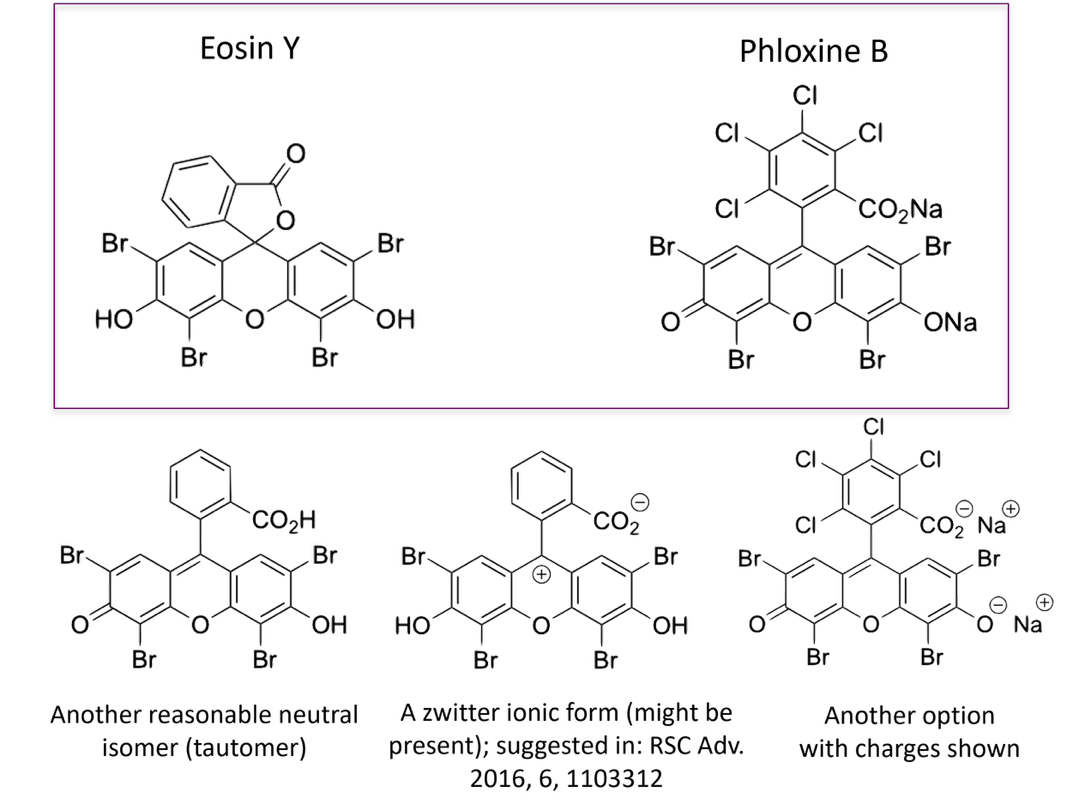
**

# **Chart 1.** Possible forms of Eos and Phlox. Most probable forms are in the box.

# **
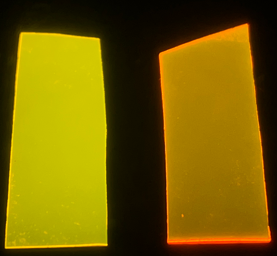
**

# **Figure SM2.** Photographs of Eos (left) and Phlox (right) in PVA film under UV illumination.

# Eos and Phlox – doped PVA films show bright luminescence emission.

# ****

# **Figure SM3.** Absorption spectra of of Eos (left) and Phlox (right) in PVA films at different dye concentrations.

# ****

# **Figure SM4.** Absorption spectra of Eos and Phlox in PVA films and Rhodamine 6G in ethanol. The excitation wavelength (510 nm) is indicated by an arrow.

# ****

# ****

# **Figure SM5.** Delayed fluorescence excitation anisotropies of Eos (top) and Phlox (bottom) in PVA films.

# ****

# ****

# **Figure SM6.** Absorption (top) and DF/RTP (bottom) spectra of the PVA film doped with both dyes (Eos and Phlox).

# The broad red luminescence emission can be achieved by combining both dyes.
